# Saxagliptin Induces β-Cell Proliferation through Increasing Stromal Cell-Derived Factor-1α *In Vivo* and *In Vitro*

**DOI:** 10.3389/fendo.2017.00326

**Published:** 2017-11-27

**Authors:** Chun-Jun Li, Bei Sun, Qian-Hua Fang, Min Ding, Yun-Zhi Xing, Li-Ming Chen, De-Min Yu

**Affiliations:** ^1^Key Laboratory of Hormones and Development (Ministry of Health), Tianjin Key Laboratory of Metabolic Diseases, Tianjin Metabolic Diseases Hospital & Tianjin Institute of Endocrinology, Tianjin Medical University, Tianjin, China

**Keywords:** DPP-4 inhibitor, β-cell, type 2 diabetes, stromal cell-derived factor-1, cell proliferation

## Abstract

Dipeptidyl peptidase-4 inhibitors, such as saxagliptin, have been reported to have beneficial effects on β-cell function, but the specific underlying mechanism remains unclear. Stromal cell-derived factor-1α (SDF-1α), a chemokine produced in multiple organs, has been considered as a crucial regulator in promoting β-cell survival. Here, we speculate that SDF-1α might mediate the effect of saxagliptin on improving β-cell function. After 12-week saxagliptin treatment in high-fat diet/streptozotocin-induced diabetic rats, significant improvement in pancreas insulin secretion capacity evaluated by hyperglycemia clamp and increased β-cell to α-cell areas ratio were observed. Saxagliptin significantly induced β-cell proliferation and upregulated the expression of proliferation-related factors including c-myc and cyclind D1 determined with western blotting from the isolated islets. The expression/activity of DPP-4 was significantly reduced and paralleled with the restoration of SDF-1α levels in the saxagliptin-treated diabetic rats, subsequently the key WNT-signaling regulators, β-catenin, and AKT were activated. However, the effect of saxagliptin inducing β-cell proliferation was attenuated when we silenced the SDF-1α receptor (CXCR4) with RNAi in INS cell lines. Collectively, our data indicate that SDF-1α mediates the protective effect of saxagliptin on β-cell proliferation, suggesting that DPP-4 inhibitors have the potential role on delaying β-cell failure and SDF-1α could be a therapeutic target of β-cell regeneration.

## Introduction

Type 2 diabetes mellitus (T2DM) is a progressive disease characterized by progressive loss of glycemic control and β-cell dysfunction (1). United Kingdom Prospective Diabetes Study reported that the β-cell function in newly diagnosed patients with T2DM had declined 50% and the β-cell function progressively declined with an estimated annual rate of approximately 4%. Therefore, saving β-cell function is most crucial for delaying the progression and treatment of T2DM. Under normal circumstances, the mass of β-cell was determined by pancreas precursor cells, and maintained a dynamic equilibrium among proliferation, apoptosis, and necrosis (2). A major objective in treatment of T2DM is to identify a therapeutic agent that can prevent the gradual decline of β-cell mass. The most challenging problem is that traditional anti-diabetic drugs have no additional effects on preventing β-cell failure except for glucose control.

Recently, oral DPP-4 inhibitors newly introduced as anti-diabetic drugs could augment endogenous active glucagon-like peptide-1 (GLP-1) levels, which promote insulin secretion of pancreatic β cells and suppress inappropriate glucagon secretion in a glucose-dependent manner (3). Positive effects of DPP-4 inhibitors on glucose lowering in T2DM have been demonstrated in multiple clinical trials (4–6). Saxagliptin, a new DPP-4 inhibitor, has been reported to improve β-cell function in clinical trials and promote islet neogenesis in rodent diabetic animals (7–10). However, the mechanisms of DPP-4 inhibitors accounting for the protective effect upon pancreatic β cells are not clearly elucidated.

Substrates of DPP-4 enzyme include numerous neuropeptides, hormones, and chemokines, and stromal cell-derived factor-1α (SDF-1α) is one of the most important substrates. SDF-1α and its receptor, CXCR4, participate in tissue repair by mediating migration of circulating stem or progenitor cells to sites of damaged tissues (11, 12). Besides, it also exhibits a feature of paracrine and anti-apoptotic effects during inflammation (13). In early stage of pancreas development, SDF-1α production in β cells might be involved in their development and remodeling, however, SDF-1α expression is suppressed when they become fully differentiated (14). Of note, when islet β-cells are injured by cytokines, streptozotocin, thapsigargin, and glucotoxicity, SDF-1α expression resembling neonatal production is reinduced to enhance both growth and longevity of β-cells (14). The mechanism that SDF-1α promotes β-cell proliferation might be related to the WNT-signaling pathway. The WNT signaling is an important regulator of controlling organismal growth and differentiation, and genes encoding WNT-signaling factors expressed in the pancreas (15). Rulifson et al. reported that WNT-signaling upregulation could stimulate proliferation of both the mouse β-cell line MIN6 and primary mouse pancreatic β-cells (16). CXCR4 strongly expressed in islet β-cells, after combining with its ligand SDF-1α, activates the downstream PI3K-AKT axis and subsequently deactivates GSK-3β and stabilizes β-catenin, which are all key signaling proteins of WNT-signaling pathway (17).

DPP-4 activity in T2DM patients was found to be inappropriately increased (18, 19). Interestingly, Omar et al. recently reported that DPP-4 expression/activity in islets of obese mice chronically fed with high-fat diet (HFD) was dramatically increased compared with the control mice (20). The protective factor, SDF-1α, which secrets from β-cells under stress circumstances to induce β-cell proliferation (14), is more likely to be degraded by abnormally elevated DPP-4 activity. Therefore, we hypothesize that saxagliptin improves β-cell function through restoration SDF-1α levels by inhibiting the elevated DPP-4 activity. In our study, rat pancreas and INS-1 cells were challenged with high glucose, followed by saxagliptin treatment. We found that saxagliptin treatment-induced β-cell proliferation and improved β-cell function *via* suppression the degradation of SDF-1α. These results, for the first time to our knowledge, point out that SDF-1α is an important factor in mediating the effect of the DPP-4 inhibitor saxagliptin on improving β-cell function.

## Research Design and Methods

### Animals and Treatments

Eight-week-old male Sprague Dawley rats (*n* = 30; HFK Bioscience Co. Ltd., Beijing, China) weighing 180–200 g were housed under a 12 h light/dark cycle and all animals had a free access to food and water. To induce T2DM models, animals were placed on an HFD (D12492, Research Diets) after 3–5 days’ acclimation, which was maintained for the duration of the study. The compositions of HFD are shown in Table 1. After 8 weeks of HFD feeding, a single low-dose STZ (30 mg/kg body weight, Sigma Chemical, USA) dissolved in sodium citrate buffer was tail-intravenous injected after an 8 h fasting. One week after STZ injection, 8 h fasting blood glucose levels of the HFD/STZ rats were measured and blood glucose that >11.1 mmol/L was confirmed diabetes. Rats with similar degree of hyperglycemia were randomly divided into two groups and were treated for 12 weeks: the saxagliptin group (*n* = 10) received saxagliptin (1 mg/kg; Bristol-Myers Squibb, Pennington, NJ, USA), and the DM group (*n* = 10) received normal saline. An age-matched control group was included and fed with regular rodent chow diet. Random blood glucose from tail bleed and body weight were monitored once every 2 weeks during the study. This study was approved by the Animal Use Committee of Tianjin Medical University (Ref. 20150902) and was conducted in compliance with the Animal Use Guidelines of the university committee.

**Table 1:** Compositions of NFD and HFD.

NFD	HFD			

Ingredient	g	kcal	g	kcal
Casein, 80 mesh	200	800	200	800
L-cystine	3	12	3	12
Corn starch	315	1,260	0	0
Maltodextrin 10	35	140	125	500
Sucrose	350	1,400	68.8	275.2
Cellulose, BW200	50	0	50	0
Soybean oil	25	225	25	225
Lard	20	180	245	2,205
Mineral max, S10026	10	0	10	0
Dicalcium phosphate	13	0	13	0
Calcium carbonate	5.5	0	5.5	0
Potassium citrate, 1 H_2_O	16.5	0	16.5	0
Vitamin mix, V1001	10	40	10	40
Choline bitartrate	2	0	2	0
FD&C yellow dye #5	0.05	0	0	0
FD&C blue dye #1	0	0	0.05	0
Total	1,055.05	4,057	773.85	4,057

*HFD, high-fat diet; NFD, normal-fat diet*.

### Hyperglycemic Clamp and Insulin Tolerance Test (ITT)

After 12-week saxagliptin treatment, the animals were fasted overnight and anesthetized with an intraperitoneal injection of 10% chloral hydrate (300 mg/kg body weight). After the anesthesia was delivered, the animals’ left carotid artery and caudal vein were catheterized. The venous catheter was used for the infusion of glucose and the arterial catheter was used for sampling (21). After 30 min of the catheters were placed in the rats, hyperglycemic clamp was initiated with a priming dose of glucose (350 mg/kg; 1 min) administered into the caudal vein and a 25% glucose solution was infused through the venous catheter at a slow rate. Blood glucose concentration was measured every 5–10 min to maintain levels at 5.5 mM above the fasting concentration by adjusting the glucose infusion rate. Arterial blood samples (~400 μL) were collected at 0, 5, 10, 60, 90, and 120 min and immediately centrifuged. Each sample was stored at −80°C for later analysis of plasma insulin using an ELISA kit (Millipore-linco EZRMI-13K). The ITT was conducted by an intraperitoneal injection of insulin (0.75 U/kg body weight, Novolin R; Novo Nordisk) after overnight fasting. Tail vein blood was measured at 0, 30, 60, and 120 min with glucometer.

### Cell Culture

INS-1 832/13 cells (kindly provided by Professor Daiqing Li, Department of Endocrinology, Metabolic Disease Hospital, Tianjin Medical University, China) were cultured with RPMI-1640 medium containing 11.0 mmol/L glucose, 10% fetal bovine serum, 100 U/mL penicillin, 100 U/mL streptomycin, 10 mmol/L glutamine, 1 mmol/L sodium pyruvate, 10 mmol/L HEPES, and 50 µm β-mercaptoethanol in an atmosphere of 5% CO_2_ at 37°C. To identify the effects of saxagliptin, cells were exposed to 30 mM glucose for 48 h with 100 nM saxagliptin. Compounds were added 1 h before the exposure to the hyperglycemic conditions and throughout the culture. To determine the specific effect of SDF-1α on β-cell proliferation, we further applied small interfering RNA (siRNA, Gene Pharma Shanghai, China) that silences CXCR4 expression followed by with/without saxagliptin treatment in high-glucose condition for 48 h. Cell lysates were collected for Western blot analysis.

### Immunohistochemistry

Pancreas tissues were fixed in 10% buffered formaldehyde at 4°C overnight and then embedded in paraffin. Four-micrometer sections applied to slides and stained with hematoxylin and eosin for light microscopic examination. Immunohistochemical methods were described previously (9). The sections in sodium citrate buffer (10 mmol/L sodium citrate containing 0.05% Tween 20, pH 6.0) were heated for 15 min at 90°C with microwave oven for antigen retrieval, and immersed in methanol for another 15 min for blocking non-specific background staining. After washing in TBS, the sections were co-incubated with anti-insulin and anti-glucagon (Sigma-Aldrich, St Louis, MO, USA) or anti-glucagon and anti-DPP-4 (Abcam, Cambridge, UK) or anti-insulin and anti-SDF-1α (Abcam, Cambridge, UK) or anti-insulin and anti-PCNA (BD Biosciences, Tokyo, Japan) primary antibodies at 4°C overnight followed by secondary antibody for 2 h at room temperature in the shade. They were then washed and finally mounted in aqueous medium with DAPI (Vectashield Mounting Medium with DAPI; Vector Laboratories). Stained sections were observed under confocal microscope (Olympus FV1000, Tokyo, Japan) and digital images were collected. For quantification purpose, commercial software (BZ Analyzer; Keyence) was used to measure the β-cell area, α-cell area, and islet area. We also counted the number of insulin-positive and PCNA-positive cells according to DAPI (nuclear) staining. Observations were from 10 sections from 3 different areas of the pancreas including a minimum of 50 islets for each group of rats. All morphometric studies were performed in a blinded fashion.

### DPP-4 Activity Assays

To measure the activity of DPP-4, we used a DPP-4 Assay Kit (Enzo Life Sciences, Farmingdale, NY, USA) with the Gly-Pro-para-nitroaniline (pNA) chromogenic substrate, according to the manufacturer’s instructions. Cells grown in six-well plates were homogenated in buffer containing 200 µL ordinary RIPA lysis (Solarbio, Beijing, China), 0.2 µL PMSF (Solarbio, Beijing, China), and 0.2 µL protease inhibitor cocktail (Boster Biological Technology, Wuhan, China). Start the assay by mixing 20 µL of lysate with 10 µL of 5 mM Gly-Pro-pNA and then read the plate record data immediately at 2 min intervals for a total of 60 min. DPP-4 activity was calculated according to the released pNA by measuring the absorbance at 405 nM with a standard curve of p-nitroanalide. The enzyme activity was defined as the amount of enzyme catalyzing the formation of p-nitroanalide/s.

### Islets Isolation and Western Blot

Islets were isolated by the Histopaque gradient solutions technique of Noordeen et al. (22) with a slight modification. In brief, after a light ether anesthesia, the animal was decapitated, bled, and the abdomen was opened. And then collagenase (1.0 mg/mL in Hanks’ balanced salt solution) was injected into the pancreatic duct (10.0 mL/rat). The distended pancreas was then incubated in a shaking water bath at 37°C in 1.0 mg/mL collagenase in Hanks’ balanced salt solution for 10 min, and the islets were recovered using Histopaque gradient solutions (5 mL of 1.119 g/L; 5 mL of 1.083 g/L; and 5 mL of 1.077 g/L). After centrifugation for 20 min at 2,500 × *g*, islets were removed from the top layer. For immunoblots, cultured INS cells and isolated islets were lysed in a buffer containing 20 mmol/L. Samples (25–50 µg protein/lane) were electrophoresed through a 4–15% precast linear gradient polyacrylamide gel, followed by a transfer of proteins to nitrocellulose membranes and then immunoblotting, as we described before (23). Rabbit polyclonal primary antibodies against Akt, phospho-Akt (Ser473), c-myc, cyclin D1, SDF-1α as well as β-catenin (active form with the mutated GSK-3β phosphorylation sites from UpState 8E7) (all from cell signaling) were used according to manufacturers’ instructions. Mouse monoclonal anti-β-actin (cell signaling) was used to evaluate the amount of protein loaded in each sample. Protein density was quantified by densitometry analysis using Image J software, and the results were presented as a ratio between the intensity of total proteins and active proteins.

### Statistical Analysis

Data are expressed as mean ± SEM. The samples in animal study were 5–10 for each group and all of the *in vitro* experiments were replicated at least three times. Statistical analysis was performed using one-way ANOVA followed by the SNK multiple comparison post-tests. Graphical were performed using GraphPad Prism 5 (GraphPad Software, San Diego, CA, USA). Statistical and data analysis were conducted using IBM SPSS software 20.0 (SPSS, Chicago, IL, USA). Values of *p* < 0.05 were considered significant differences.

## Results

### Effects of Saxagliptin on Body Weight, Blood Glucose, and Islet Function in HFD/STZ-Induced Diabetic Rats

**Figure 1:**
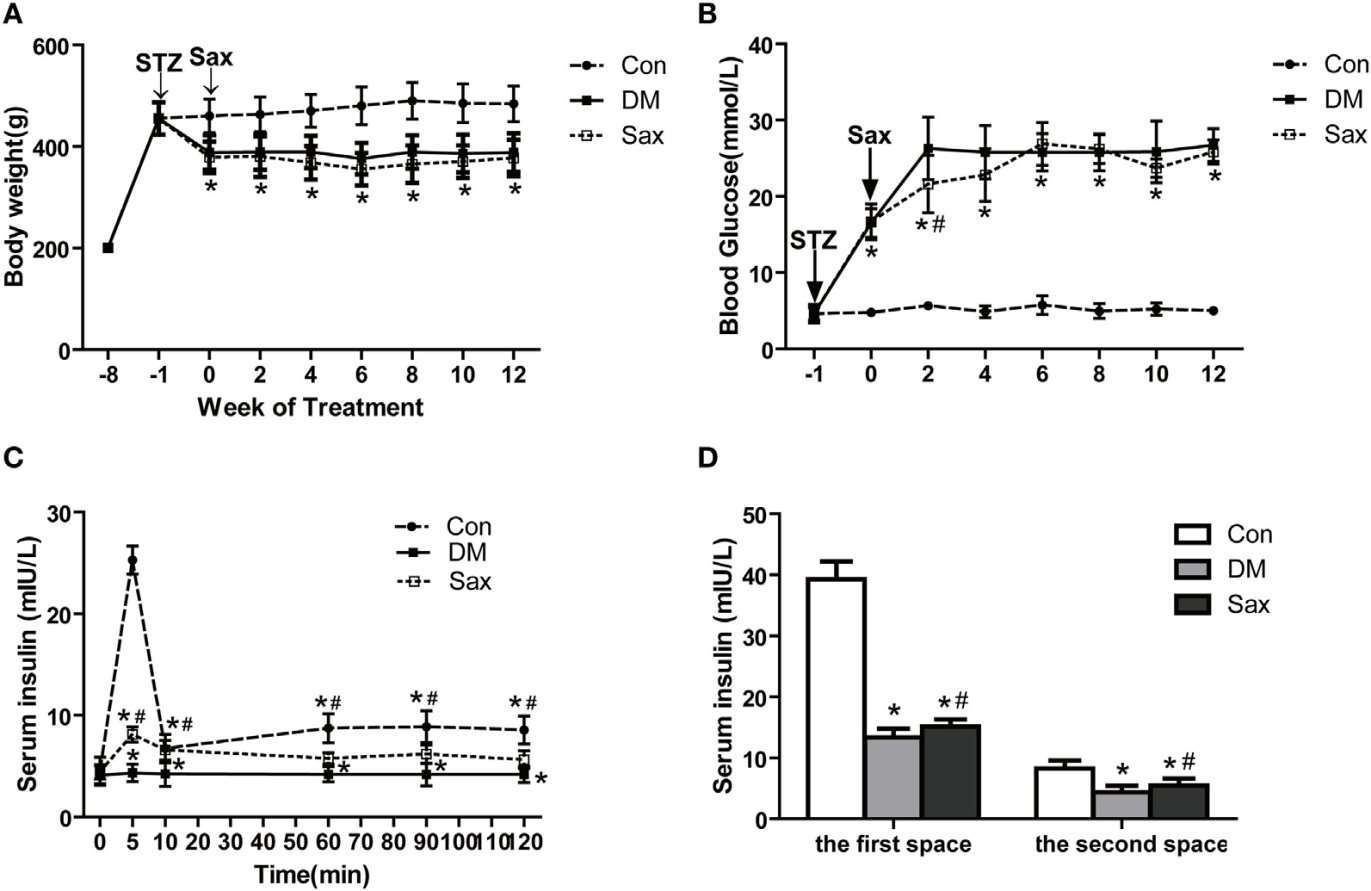
Effects of saxagliptin on body weight, blood glucose, and islet function in high-fat diet/STZ-induced diabetic rats. Body weight was measured every 2 weeks **(A)**. Blood glucose levels were measured every 2 weeks (*n* = 7–10 in each group) **(B)**. Insulin secretion levels during hyperglycemic clamp (*n* = 3 in each group) **(C)**. The first and second phase of insulin secretion of each group **(D)**. The first-phase insulin secretion levels were the sum of the insulin levels of 5 and 10 min after venous glucose loading. Similarly, the second-phase insulin secretion levels were the average plasma insulin levels of 60, 90, and 120 min after venous glucose loading. Data were reported as mean ± SEM. *p < 0.05 compared with the control group, ^#^*p* < 0.05 compared with DM group. Con, control group; DM, diabetes group; Sax, saxgliptin group.

Sprague Dawley rats were induced with the HFD for 12 weeks and then administrated with STZ to induce diabetes, and after 1 week of STZ injection, treated with saxagliptin for another 12 weeks. As shown in Figure 1A, body weight was similar among the three groups before STZ administration. DM rats and saxagliptin-treated DM rats became statistically thinner than control rats fed the chow diet from the start of saxagliptin treatment (indicated 0 week) to 12 weeks (Figure 1A). However, during the intervention period, there was no difference on body weight between the DM group and the Sax group (Figure 1A). At the start of saxagliptin treatment, the average blood glucose levels in diabetic rats were significantly higher than that in control rats. After 2 weeks of saxagliptin administration, saxagliptin-treated DM rats began to show a significant reduction of blood glucose compared with the vehicle-treated DM rats. Unexpectedly, the blood glucose in diabetic rats did not show any significant glucose lowering effect with saxagliptin treatment compared with vehicle treatment DM rats from 4 to 12 weeks (Figure 1B).

A hyperglycemic clamp was performed on rats subjected to overnight fasting to determine insulin secretion capacity of different groups at the end of 12-week saxagliptin intervention. During the hyperglycemic clamp, blood glucose levels were kept at 5.5 mM above the baseline, compared with the control rats, diabetic rats had significantly lower insulin secretion levels at 5 and 10 min, which are regarded as the first-phase insulin secretion after venous glucose loading. As expected, diabetic rats receiving saxagliptin treatment exhibited a higher first-phase insulin secretion levels compared with the diabetic rats treated without saxagliptin. The second-phase insulin secretion levels, that is, determined by the average plasma insulin levels at 60, 90, and 120 min after venous glucose loading, were significantly reduced in the diabetic rats compared with the control rats. Similarly, saxagliptin treatment significantly enhanced the second-phase insulin secretion levels compared with the vehicle-treated diabetic rats (Figures 1C,D). During the ITT test, administration of insulin led to a significant decrease of blood glucose levels both in the saxagliptin group and the diabetic group compared with the baseline, but the decrease trends were significantly higher in the Sax group compared with the DM group (Figure S1A in Supplementary Material). The area under the curve showed significantly improved insulin sensitivity in the saxagliptin-treated diabetic rats compared with the diabetic rats treated with vehicle (Figure S1B in Supplementary Material).

### Protective Effect of Saxagliptin on Islet Morphology in HFD/STZ-Induced Diabetic Rats

**Figure 2:**
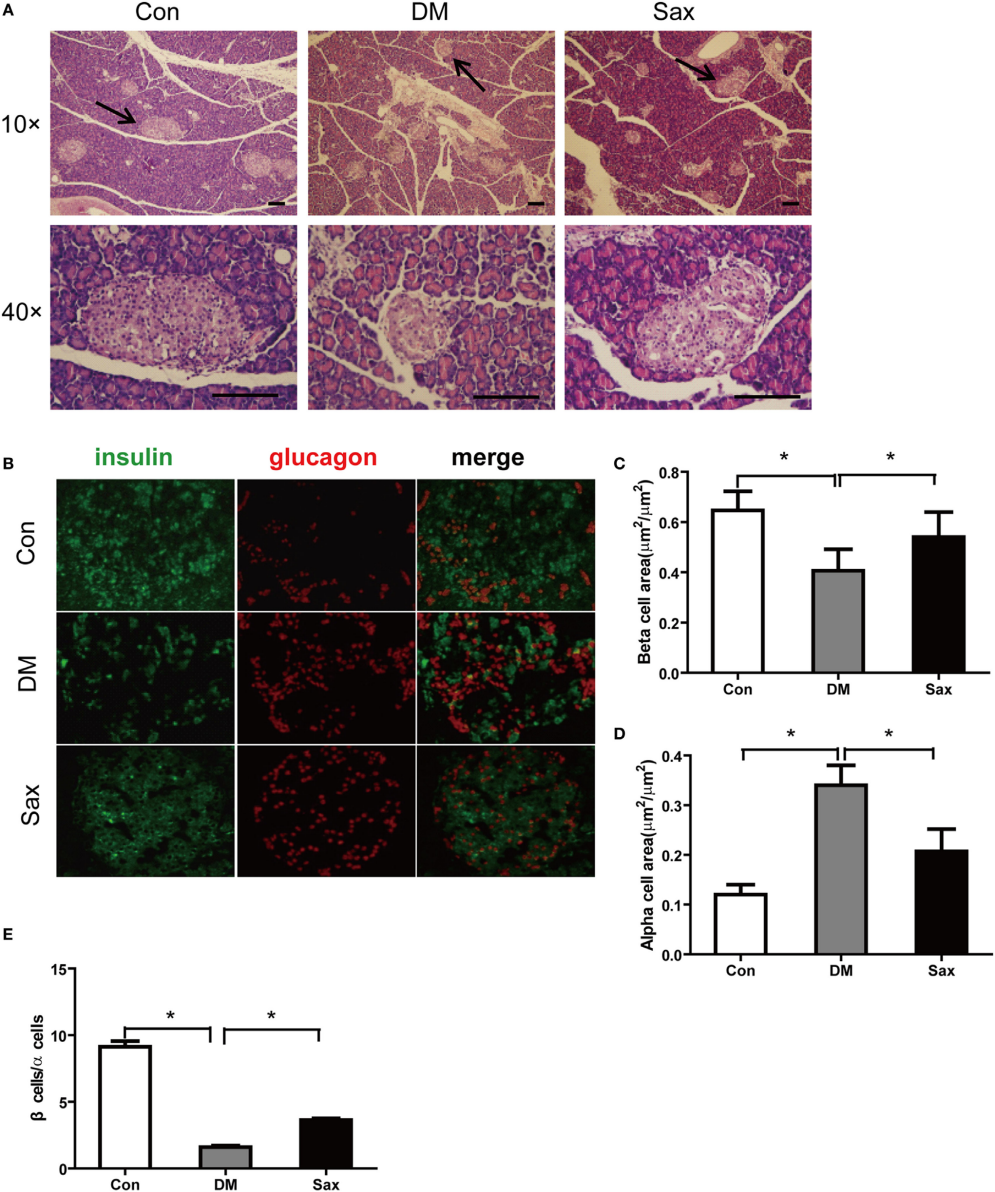
Effects of saxagliptin on islet morphology. Representative pancreas sections were stained with hematoxylin and eosin **(A)** or labeled with anti-insulin antibody (green) or anti-glucagon antibody (red) **(B)**. The graphs on the right represent the average percentage of insulin-positive area **(C)**. Glucagon-positive cell area **(D)**. β-cells to α-cells number ratio **(E)**. *n* = 4 for each group. Data were reported as mean ± SEM. Con, control; DM, diabetes; Sax, saxgliptin. *p < 0.05 compared with the control group and Sax group.

Compared with the Con group, rats in the DM group showed abnormal islet morphology, as evidenced by disarray of islet architecture and irregular islet boundaries, however, following 12-week saxagliptin treatment partially restored pancreatic islet architecture (Figure 2A). Double immunofluorescence staining with antibodies against insulin and glucagon showed that the insulin-positive β-cell area (green) was strikingly reduced and glucagon-positive α-cell area (red) was markedly increased with a trend of centered distribution in the diabetic rats compared with the control rats (Figures 2B–D). However, saxagliptin treatment significantly increased the insulin-positive β-cell area and decreased glucagon-positive α-cell area compared with the vehicle-treated diabetic rats. The β cells to α-cells ratio was also greater in the Sax group compared with the DM group (Figure 2E). These results indicated that DPP-4 inhibition with saxagliptin not only increased β-cell numbers but also suppressed α-cell centered expansion in HFD/STZ-induced diabetic rats.

### Saxagliptin Increased β-Cell Proliferation in HFD/STZ-Induced Diabetic Rats

**Figure 3:**
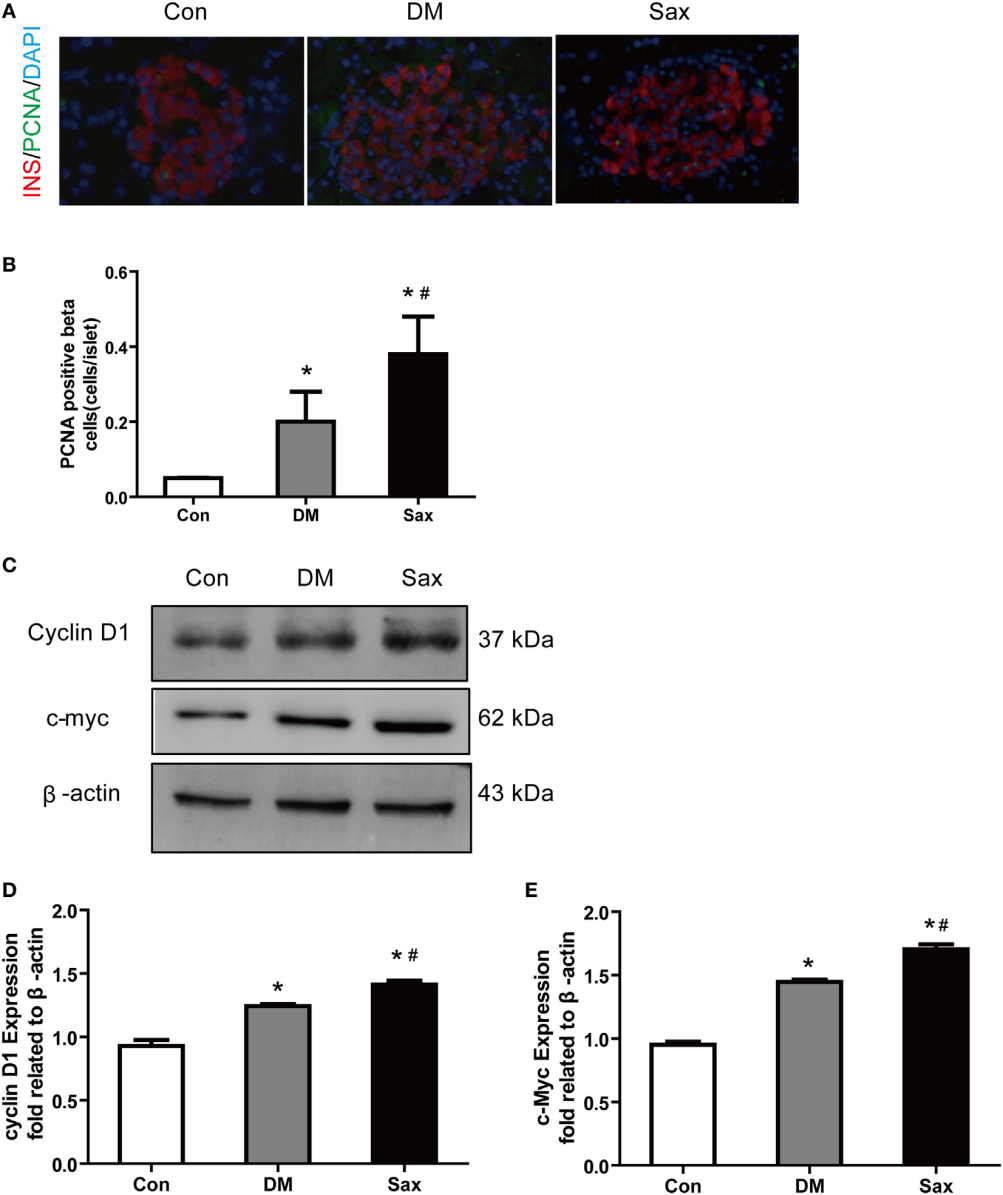
Effects of saxagliptin on β-cell proliferation in high-fat diet/STZ-induced diabetic rats. Representative pancreas sections of PCNA and insulin double immunofluorescence staining merged images from con, DM, and Sax group, insulin (red), PCNA (green), DAPI (blue) **(A)** and the positive β-cell proliferation numbers **(B)**. Western blot analysis for protein expression of c-myc and cyclin D1 in isolated islets of rats **(C)**. Densitometric analysis of indicated protein expression **(D,E)**. *n* = 4 rats for each group. Data were reported as mean ± SEM. *p < 0.05 compared with the control group, ^#^*p* < 0.05 compared with DM group. Con, control; DM, diabetes; Sax, saxgliptin.

Tissue injury usually induces proliferation process which subsequently activates developmental programs. To test this possibility, we performed PCNA and insulin double immunofluorescence staining to examine β-cell proliferation and used western blot method to test the proliferation-related protein expression including c-myc and cyclin D1 from isolated islets (24, 25). As shown in Figures 3A,B, PCNA and insulin double positive cells of islets in the Sax group were remarkably increased than that in the DM group. The western blot results of two proliferation-related protein from isolated islets, c-myc, and cyclin D1, further confirmed PCNA and insulin double immunostaining data (Figures 3C–E). The protein expression of c-myc and cyclin D1 had an increased trend in the islets of diabetic rats than those of normal rats. In addition, saxagliptin treatment significantly augmented c-myc and cyclin D1 expression compared with the diabetic rats treated with vehicle. These results indicate that HFD/STZ exposure resulted in β-cell loss and subsequently activated the β-cell proliferation. Saxagliptin treatment intensified the protective mechanism against islet injury by increasing β-cell proliferation.

### Saxagliptin Strengthened the SDF-1α/Akt/β-Catenin Pathway by Inhibition the Elevated DPP-4 Expression/Activity in HFD/STZ-Induced Diabetic Rats

**Figure 4:**
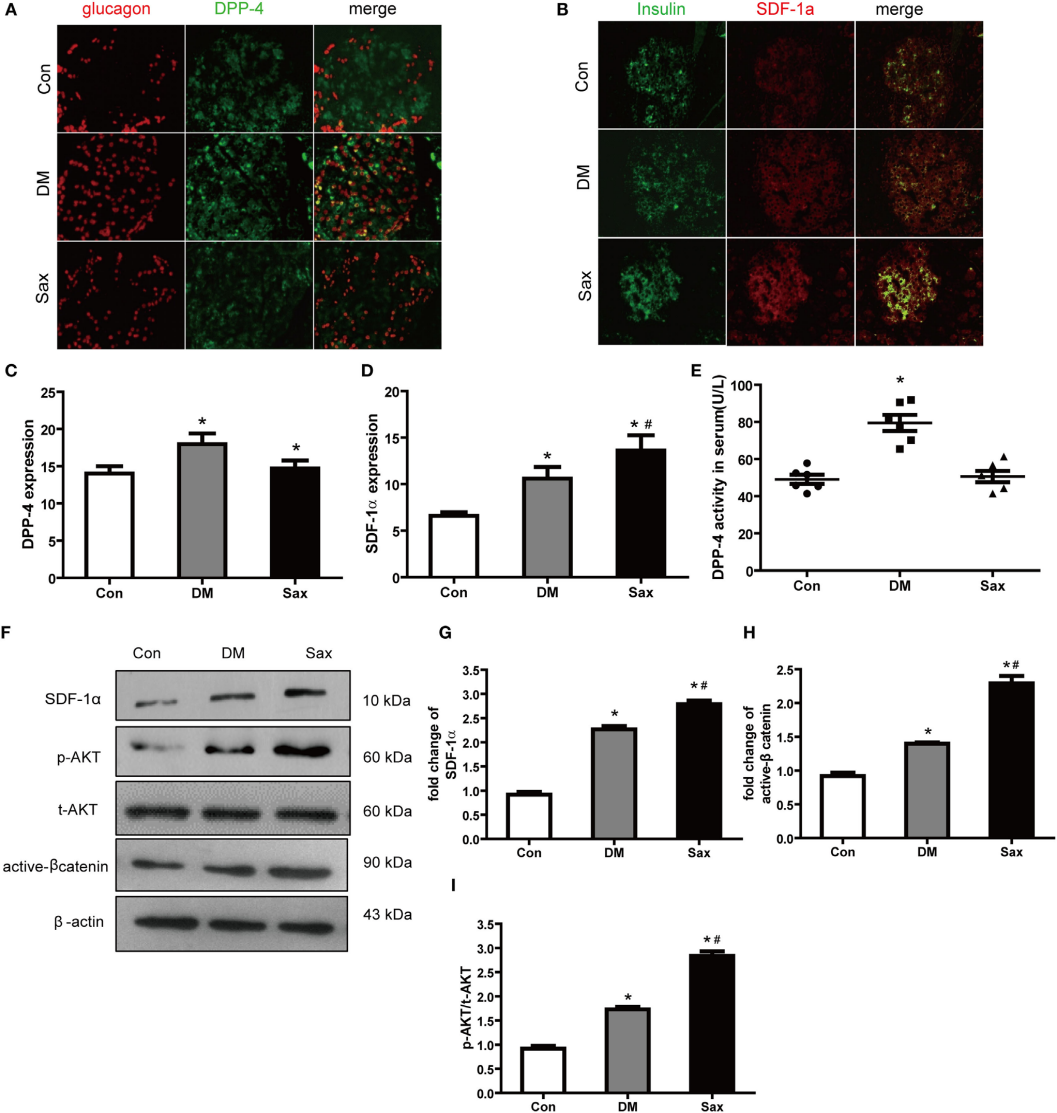
Saxagliptin strengthened the stromal cell-derived factor-1α (SDF-1α)/Akt/β-catenin pathway in high-fat diet/STZ-induced diabetic rats. Double immunofluorescence images of pancreatic islets stained with antibodies to glucagon (red) and DPP-4 (green) **(A)**. Double immunofluorescence images of pancreatic islets stained with antibodies to insulin (green) and SDF-1α (red) **(B)**, ×400. The graphs on the right represent the average of DPP-4 expression **(C)** and SDF-1α expression **(D)** in rat islets. DPP-4 enzyme activity in serum from each group **(E)**. Western blot analysis for active β-catenin, SDF-1α, and phosphorylation of Akt (p-AKT) in isolated islets of different groups **(F)**. Densitometric analysis of indicated protein expression **(G–I)**. *n* = 4 for each group. Data were reported as mean ± SEM. *p < 0.05 compared with the control group, ^#^*p* < 0.05 compared with DM group. Con, control; DM, diabetes; Sax, saxgliptin.

As we hypothesized saxagliptin protect β-cell function by inhibition DPP-4 expression/activity and preventing SDF-1α degradation, we detected DPP-4 and SDF-1α expression of islets with double immunofluorescence staining. In the control rats, DPP-4 was readily detected in islets with predominately expression in the β-cell area (Figure 4A). In the diabetic rats, the DPP-4 expression of islets was stronger than that in the control rats. Interestingly, saxagliptin treatment significantly reduced the DPP-4 expression of islets compared with the vehicle-treated diabetic rats. We also detected the DPP-4 activity in serum and found that saxagliptin dramatically decreased the diabetes-enhanced DPP-4 activity in HFD/STZ-induced diabetic rats (Figures 4C,E). The immunohistochemistry revealed robust SDF-1α restricted to the β-cell area too, and high glucose induced a slightly higher expression of SDF-1α in the diabetic rats than those of normal rats (Figures 4B,D,F). Of note, saxagliptin treatment resulted in an approximately 1.5-fold higher SDF-1α level of islets compared with the DM group Figure 4G. Akt and β-catenin are key factors of SDF-1α inducing WNT signaling and ultimately induce β-cell proliferation (17). To test whether the effects of saxagliptin on β-cell proliferation are mediated by the two factors, the phosphorylation of Akt (p-AKT) and active β-catenin (unphosphorylated on Ser-33 and Ser-37) were examined from the isolated islets. We found that p-Akt in the DM group was slightly increased in comparison with the Con group, in accordance with the increased SDF-1a by saxagliptin in HFD/STZ-induced diabetic rats, and p-Akt was also remarkably increased compared with the DM group (Figures 4F,I). And active β-catenin expression was slightly higher in the isolated islets of diabetic rats than that of control rats. After 12-week saxagliptin treatment, active β-catenin level was further increased significantly (Figures 4F,H). Taken together, these data revealed that saxagliptin treatment could increase SDF-1α *via* DPP-4 inhibition and a concomitant activation of its downstream factors p-Akt and active β-catenin in pancreas of HFD/STZ-induced diabetic rats.

### Saxagliptin Increased β-Cell Proliferation Depends on the SDF-1α/CXCR4 Pathway in Cultured INS-1 Cells

Previous study had shown that INS-1 cells expressed DPP-4 (26), we detected the effect of saxagliptin on DPP-4 activity in INS-1 cells. In line with *in vivo* study, 100 nM saxagliptin intervention reduced DPP-4 activity and expression compared with untreated high-glucose group, and a concomitant increase of SDF-1α protein levels (Figures S2A–D in Supplementary Material).

**Figure 5:**
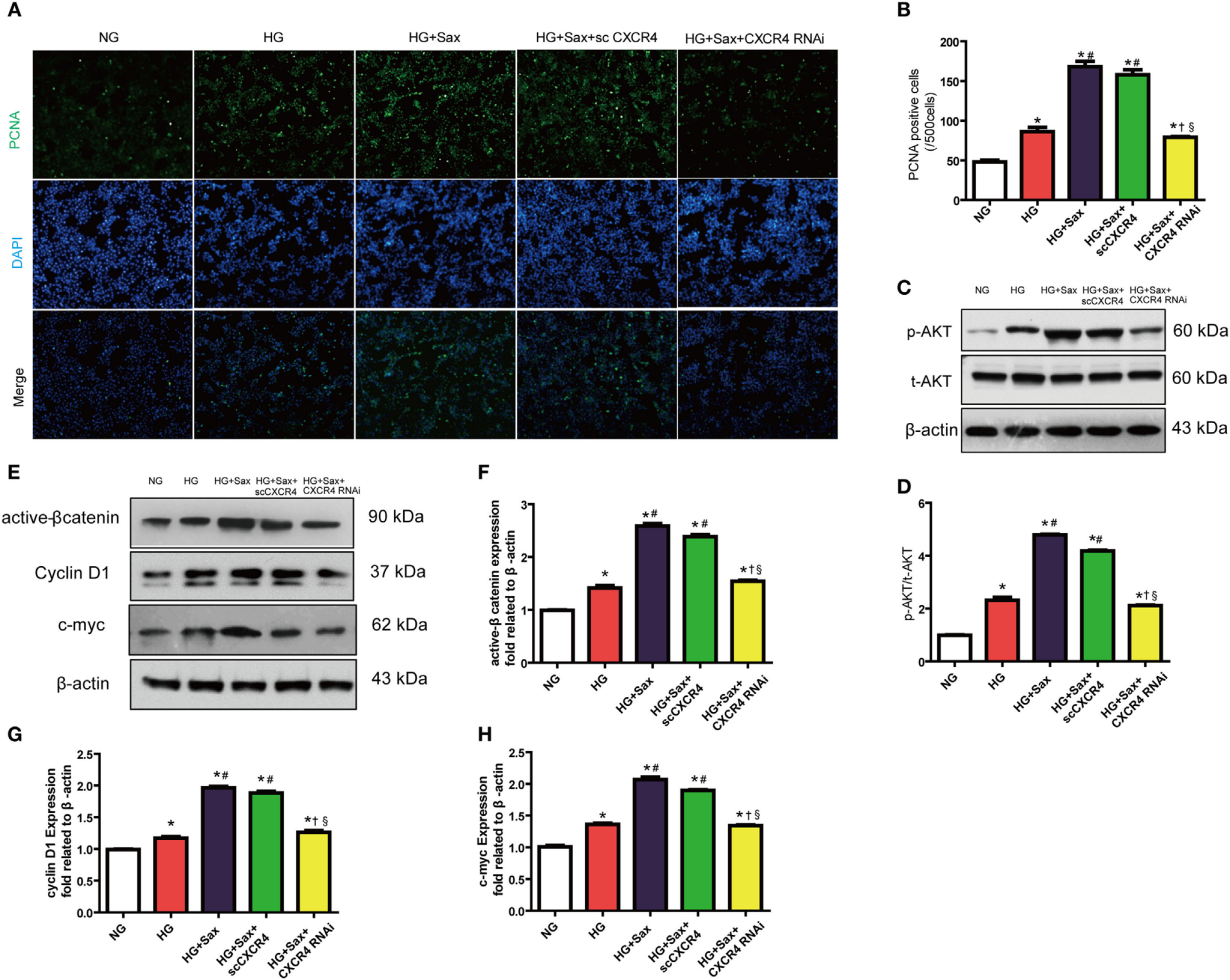
Saxagliptin-induced β-cell proliferation depends on the stromal cell-derived factor-1α/CXCR4 pathway in INS cells. Representative images showing PCNA-positive cells in cultured INS-1cells from different conditions **(A)** and the positive cell number analysis **(B)**. Western blot analysis for protein expression of p-AKT in pancreas of rats **(C)**. Densitometric analysis of t-AKT protein expression relative to t-AKT is shown **(D)**. Western blot analysis for indicated protein expression from different conditions **(E)**. Densitometric analyses of indicated protein expression relative to β-actin levels are shown **(F–H)**. Data were reported as mean ± SEM (*n* = 3). *p < 0.05 compared with the NG group, ^#^*p* < 0.05 compared with HG group, ^†^*p* < 0.05 compared with HG + Sax group; ^§^*p* < 0.05 compared with HG + with HG + Sax + scCXCR4 group. NG, normal glucose; HG, high glucose; Sax, saxagliptin; scCXCR4, scramble CXCR4 RNAi; p-AKT, phosphorylation of Akt; t-AKT, total AKT.

To further illuminate whether the effects of saxagliptin treatment-induced β-cell proliferation are mediated by SDF-1α-related pathway, we performed *in vitro* studies after silencing CXCR4, a high-selective SDF-1α receptor, using CXCR4 small interference RNA in INS-1 cell lines (Figure S3 in Supplementary Material). In accordance with increased β-cell proliferation by saxagliptin treatment in HFD/STZ-induced diabetic rats, PCNA immunofluorescence staining revealed that 100 nM saxagliptin could increase cell proliferation in INS-1 cells (Figures 5A,B). In addition, saxagliptin increased the p-AKT and active β-catenin protein levels, paralleled with the increase of c-myc and cyclin D1 protein expression (Figures 5C–H). After silencing CXCR4 using RNAi, the above protective effects of saxagliptin were attenuated sharply (Figures 5A–G). Collectively, these data indicate that the SDF-1α/CXCR4 pathway might be responsible for saxagliptin-induced β-cell proliferation.

## Discussion

β-Cell dysfunction represents the core defect of T2DM, therefore, the primary goal of the present study was to evaluate whether treatment with DPP-4 inhibitor saxagliptin could initiate the protection mechanism on inducing β-cell proliferation. We demonstrated that saxagliptin treatment improved pancreas insulin secretion capacity and increased β-cell area through increasing SDF-1α levels in the HFD/STZ-induced diabetic rats. More importantly, we first reported that the expression of DPP-4 in the islets of HFD/SZT-induced diabetic rats was significantly increased while saxagliptin treatment could alleviate the adverse change. The protective effect of saxagliptin might be mediated by increasing SDF-1α to activate the downstream key effectors Akt and β-catenin and proliferation correlated protein, c-myc, and cyclin D1. In INS-1 cells, saxagliptin significantly reduced the activity of DPP-4 and increased β-cell proliferation, but the protective effect of saxagliptin was attenuated when blocked the SDF-1α pathway with CXCR4 siRNAs. Our data suggest that DPP-4 inhibitor saxagliptin could promote pancreatic β-cell proliferation through SDF-1α/CXCR4 pathway.

Numerous studies have reported that DPP-4 inhibitors played an important role in regulating the differentiation from the duct cells (27), neogenesis and apoptosis of the pancreatic β-cells, and shown favor influence on blood glucose control and β-cell function in clinical (5, 6) and in rodent studies (8, 27). Unexpectedly, in this study, we did not observe obvious hypoglycemic effect of saxagliptin treatment on HFD/STZ-induced diabetic rats. This might be resulted from the rat pancreas seriously damaged by STZ injection even though we adopted a low-dose STZ of 30 mg/kg body weight, a conventional dose that is widely used in other studies. We speculated that the β-cell protective effect on saxagliptin may not be enough to compensate for the STZ-induced sever pancreas injury in the short-term intervention (28–30). Anyway, we found the improved β-cell function evaluated by hyperglycemia clamp and increased β-cell to α-cell areas ratio observed by double immunohistochemistry fluoresce staining. But it would be better if we assayed the glucose stimulated insulin secretion in the isolated islets to support out results. Therefore, long-term saxagliptin intervention and detailed *ex vivo* study are need to test the β-cell protective effect through initiating the proliferation process in the future. Nonetheless, in line with previous experiments, we found that given diabetic rats 12-week saxagliptin treatment could significantly improve insulin secretion capacity in both the first and second phase at hyperglycemic clamp and β-cell area of islets in the saxagliptin group had a greater increase compared with the DM group. However, the specific mechanism that DPP-4 inhibitors protect β-cell function is still unclear. Currently, researchers are more likely attribute to the beneficial effect of DPP-4 inhibitors on diabetes to GLP-1 (31, 32), but studies showed that other substrate such as SDF-1α has a function on protection β-cell survival as well.

Liu et al. reported that SDF-1 produced in the β-cells of the neonatal but not the adult mouse pancreas, while under stressed circumstances injured β-cells would reproduce SDF-1α in order to enhance both growth and longevity of β-cells (14). Furthermore, transgenic mice overexpressing SDF-1α with in β-cells (RIP-SDF-1 mice) could resistant to STZ-induced β-cell apoptosis and diabetes through activating the Akt protein kinase, while SDF-1α receptor antagonist AMD3100 induced apoptosis in MIN6 β-cells (33), suggesting that upregulation of SDF-1α in islets may represent a defense system against β-cell injury. Similarly, we observed that high glucose increased SDF-1α protein levels both *in vivo* and *in vitro* studies and saxagliptin treatment further increased the level of SDF-1α due to inhibiting the expression/activity of DPP-4. In INS-1 cells, there was a relative high level of SDF-1α expression in the 100 nM saxagliptin group, indicating that the chemokine SDF-1α might act like the cytokines inducing its own expression in β-cells by an autocrine mechanism (14) while the pancreas in the Con group had a low expression of SDF-1α.

Traditionally, the WNT-signaling pathway is correlated with regulation of pancreas development and differentiation and emerging results from different groups identified the WNT-signaling pathway as a crucial regulator of prenatal β-cell development (34) and postnatal β-cell functions (16, 35) in recent years. Although the expression of important components of WNT-signaling pathway have been demonstrated in adult pancreatic β-cells (15), very little is known about how these endogenous molecules are regulated in β-cells. Recent studies found that SDF-1α could activate WNT signaling in rat neural progenitor cells (36) or islet β-cells (17) and then enhance cell survival through PI3K-AKT axis, suppression of GSK-3β, and stabilization of β-catenin. The functional role of β-catenin in SDF-1α mediated survival and cytoprotection effect of β-cells was testified by knockdown of β-catenin with siRNAs. The result showed that siRNAs antagonized the SDF-1α mediated inhibition of thapsigargin-induced β-cell apoptosis, indicating that WNT signaling is obligatory to the prosurvival effects of SDF-1α (37). Our finding firstly reported here that enhanced expression of SDF-1α in rat pancreas and INS-1 cells activated WNT and β-catenin signaling pathway by p-AKT, and the effect of saxagliptin-induced β-cell proliferation was attenuated when we silenced the SDF-1α receptor (CXCR4) with RNAi intervention.

Evidence suggests that pancreatic β-cell proliferation, rather than stem-cell differentiation, is the primary mechanism for maintaining postnatal β-cell mass (38, 39). Although the rate of replication is very slow at baseline, it can be stimulated again under some circumstances. In this study, we found that saxagliptin treatment increased the c-myc and cyclin D1 protein expression, both of them are related to cell proliferation (40). And these results were also confirmed by the PCNA and insulin double immunohistological staining of rat pancreas and INS-1 cells with PCNA staining. Kushner et al. reported that D-type cyclin knockdown mice developed life-threatening diabetes in 3-month-old after birth. Thus, cyclin D1 was essential for β-cell expansion in adult mice (24). The expression level of c-myc is tightly correlated to cell proliferation. On one hand, Myc could directly induce genes that are critical to cell cycle; on the other hand, Myc is able to hyperactivate cyclin/Cdk and antagonizes the activity of cell cycle inhibitors as p21 and p27 (41). C-myc overexpression can drive proliferation without inducing cell death in rats and human β-cells (25), and c-myc expression in pancreatic islet is upregulated during pregnancy so the β-cells will have a compensated proliferation (42).

In conclusion, we demonstrated that DPP-4 inhibitor saxagliptin improves β-cell function and increases pancreatic β-cell area by directly enhancing cell proliferation, the mechanism of which may be related to inhibition of the degradation of SDF-1α and then activating the WNT-signaling pathway. In addition, our results provide a novel theoretical basis for the clinical application of saxagliptin, which is a promise agent for T2DM therapy with disease-modifying properties and that SDF-1α might be a therapeutic target of inducing β-cell regeneration.

## Ethics Statement

This study was carried out in accordance with the Animal Use Guidelines of the university committee. The protocol was approved by the Animal Use Committee of Tianjin Medical University.

## Author’s Note

Parts of this study were presented in poster form (2072-p) at the 76th Scientific Sessions of the American Diabetes Association, New Orleans, 10–14 June 2016.

## Author Contributions

L-MC and D-MY conceived the project. C-JL and BS performed experiments and wrote the manuscript. Q-HF, MD, and Y-ZX participated in part of the work. All authors read and approved the final manuscript and declare no competing financial interests.

## Conflict of Interest Statement

The authors declare that the research was conducted in the absence of any commercial or financial relationships that could be construed as a potential conflict of interest.
