# Supplementary material for: Saxagliptin Induces β-Cell Proliferation through Increasing Stromal Cell-Derived Factor-1α In Vivo and In Vitro
Source: Front Endocrinol (Lausanne). 2017 Nov 27;8:326. doi: 10.3389/fendo.2017.00326 (PMC5711777; doi:10.3389/fendo.2017.00326)
Supplement: Supplementary file 1 [file data_sheet_1.docx]

Supplementary Material

Saxagliptin Induces β-cell proliferation through increasing stromal cell-derived factor-1α in vivo and in vitro

Chun-Jun Li^1^, Bei Sun^1^, Qian-Hua Fang, Min Ding, Yun-Zhi Xing, Li-Ming Chen* and De-Min Yu*

Key Laboratory of Hormones and Development (Ministry of Health), Tianjin Key Laboratory of Metabolic Diseases, Tianjin Metabolic Diseases Hospital & Tianjin Institute of Endocrinology, Tianjin Medical University, 300070 Tianjin, China

^1^Chun-Jun Li and Bei Sun contributed equally to this study.

*Correspondence to: Pro. Li-Ming Chen, e-mail: [xfx22081@vip.163.com](mailto:xfx22081@vip.163.com) or Pro De-Min Yu, e-mail: [yudemintij@126.com](mailto:yudemintij@126.com)


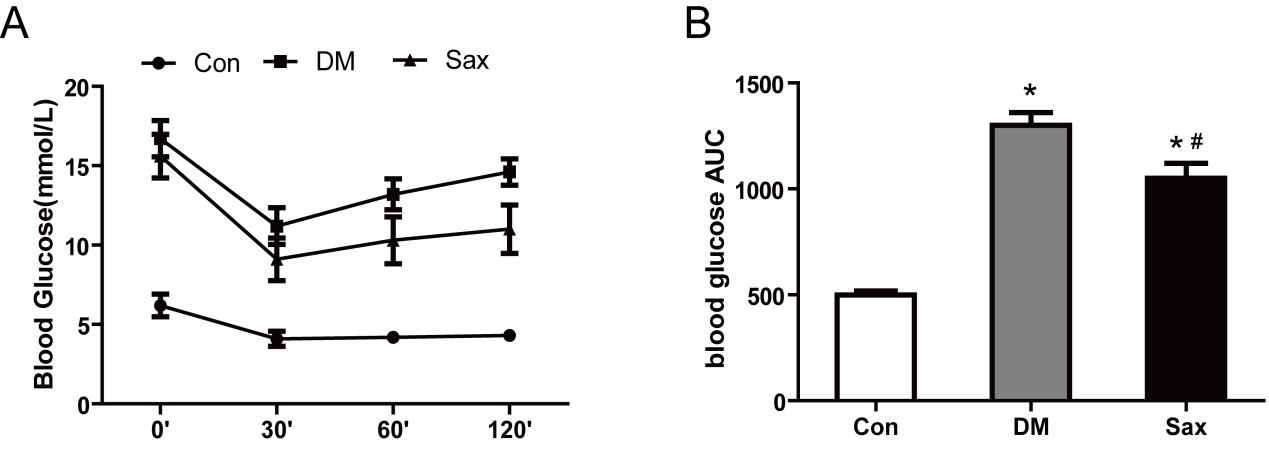


Supplemental Fig 1. Effects of saxagliptin on insulin tolerance test in HFD/STZ induced diabetic rats. ITT after saxagliptin treatment (A), area under the ITT curve (B). *p < 0.05 compared with the control group, ^#^p < 0.05 compared with DM group. Con, control; DM, diabetes; Sax, saxgliptin.


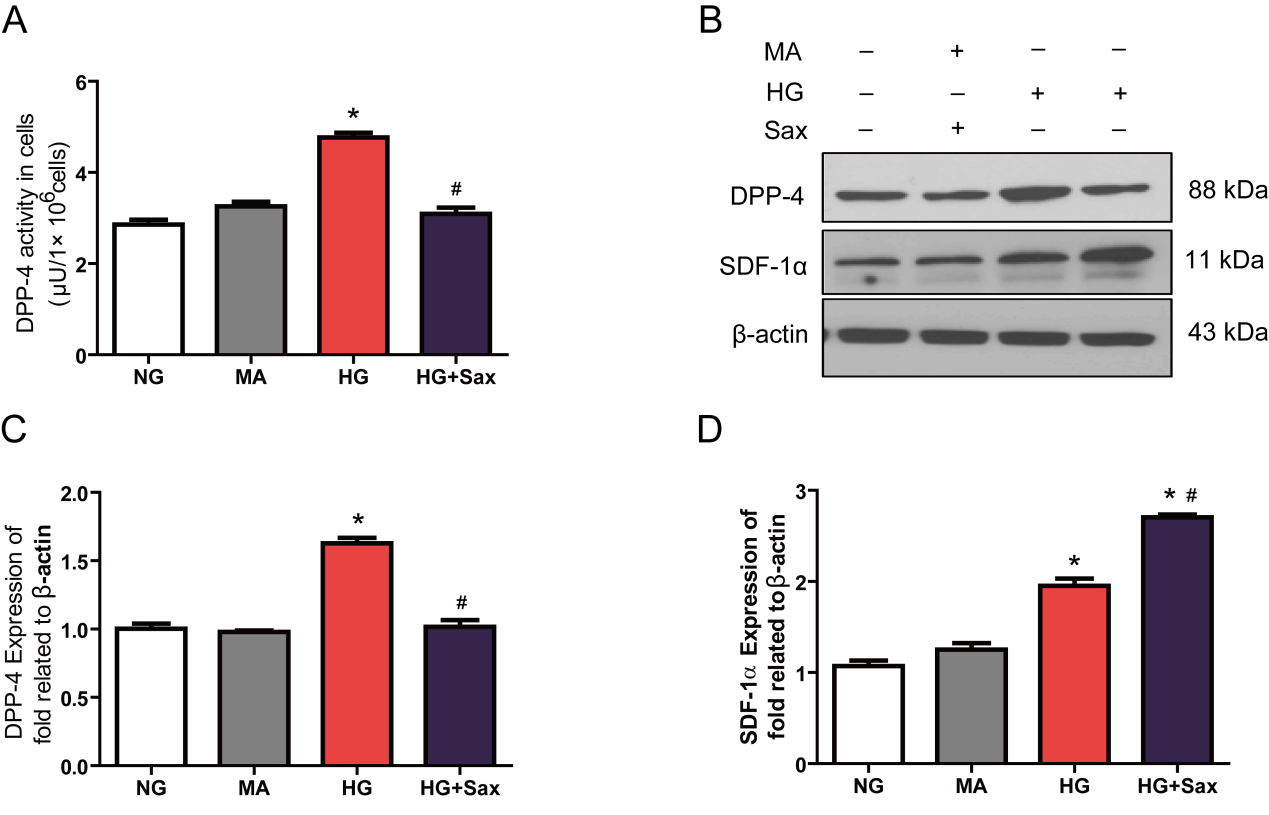


Supplemental Fig 2. Effects of saxagliptin treatment on DPP-4 activity/expression and SDF-1α protein levels in INS cells. DPP-4 activity measurements in INS cells (A). Western blot analysis for DPP-4 and SDF-1α protein expression in INS cells (B). Quantification of DPP-4 and SDF-1α expression (C and D) (n = 4). Data were reported as mean ± SEM (n=3). *p < 0.05 compared with the NG group, ^#^p < 0.05 compared with HG group, †P<0.05 compared with MA group. NG, normal glucose; MA, mannitol; HG: high glucose; Sax, saxagliptin.


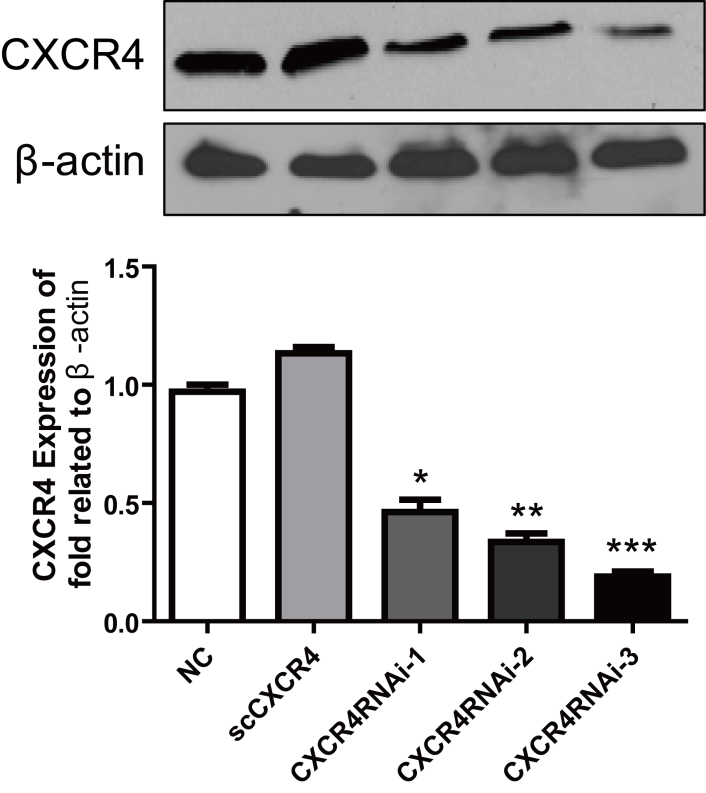


Supplemental Fig 3. Effects of transfection with CXCR4 siRNA in INS cells. Relative protein expression levels of CXCR4 were analyzed by Western Blotting and Quantification of CXCR4 expression. The results are expressed as mean ± SEM, *P < 0.05; **P < 0.01.
